# Deprescribing drugs with anticholinergic effects in older patients with increased risk of dementia in the multicomponent intervention study AgeWell.de

**DOI:** 10.1002/bcp.70194

**Published:** 2025-08-15

**Authors:** Laura K. Lepenies, Hanna M. Seidling, Alexander Pabst, Melanie Luppa, Juliane Döhring, Martin Williamson, Thomas Frese, Jochen Gensichen, Wolfgang Hoffmann, Hanna Kaduszkiewicz, Hans‐Helmut König, Jochen René Thyrian, Birgitt Wiese, Steffi G. Riedel‐Heller, David Czock

**Affiliations:** ^1^ Medical Faculty Heidelberg/Heidelberg University Hospital, Internal Medicine IX – Department of Clinical Pharmacology and Pharmacoepidemiology Heidelberg University Heidelberg Germany; ^2^ Medical Faculty Heidelberg/Heidelberg University Hospital, Cooperation Unit Clinical Pharmacy Heidelberg University Heidelberg Germany; ^3^ Institute of Social Medicine, Occupational Health and Public Health (ISAP) Leipzig University Leipzig Germany; ^4^ Institute of General Practice University of Kiel Kiel Germany; ^5^ Institute of General Practice and Family Medicine Martin‐Luther‐University Halle‐Wittenberg Halle Saale Germany; ^6^ Institute of General Practice and Family Medicine LMU University Hospital, LMU Munich Munich Germany; ^7^ German Center for Neurodegenerative Diseases (DZNE), Site Rostock/Greifswald Greifswald Germany; ^8^ Institute for Community Medicine, University Medicine Greifswald Greifswald Germany; ^9^ Department of Health Economics and Health Service Research University Medical Centre Hamburg‐Eppendorf Hamburg Germany; ^10^ Faculty V: School of Life Sciences University of Siegen Siegen Germany; ^11^ MHH Information Technology – Science and Laboratory Hannover Medical School Hannover Germany

**Keywords:** anticholinergic drugs, dementia, deprescribing, lifestyle intervention, randomized controlled study

## Abstract

**Aim:**

Drugs with anticholinergic effects are often considered as potentially inappropriate medications, especially for older patients, and deprescribing such drugs may improve cognitive function. The aim was to investigate the effectiveness of counselling on drug risks as part of a multimodal intervention to prevent cognitive decline.

**Methods:**

The AgeWell.de study, a multi‐centre, cluster‐randomized controlled study, was conducted in 123 German general practices between June 2018 and January 2022. The study included a multicomponent intervention programme for patients at increased risk of dementia, delivered over a 2‐year period. As part of the medication optimisation intervention, patient data and medication records were screened to identify medication risks and provide recommendations to general practitioners.

**Results:**

In total, 808 patients with complete data were included in the present analysis (intervention group = 374, control group = 434). At baseline, 132 (16.8%) patients had at least one anticholinergic prescription. After 2 years, approximately one‐third of these patients no longer received drugs with anticholinergic effects. There were no significant differences between the intervention and control groups, with 67.6% and 72.1%, respectively, continuing to take drugs with anticholinergic effects (*P* = 0.57). Patients reported anticholinergic symptoms more frequently when taking any medication (5.0% *vs* 33.8%), and even more so when taking drugs with anticholinergic effects (56.1%). Deprescribing of all drugs with anticholinergic effects was non‐significantly higher in patients who reported at least one anticholinergic symptom compared to patients without any anticholinergic symptoms (58.6% *vs* 54.1%).

**Conclusion:**

The medication optimisation intervention did not entail significant differences in anticholinergic deprescribing between the groups.

**What is already known about this subject?**

- Drugs with anticholinergic effects are associated with cognitive decline and adverse effects, especially in older patients.
- Deprescribing such drugs may improve cognitive function, but there is limited evidence on effective strategies for deprescribing, particularly in the context of other intervention strategies.

**What this study adds**

- This study examined the extent to which deprescribing drugs with anticholinergic effects is prioritized in the context of other potentially beneficial interventions to improve care for older patients at risk of cognitive decline.
- Counselling on drugs with anticholinergic effects as part of the multimodal intervention in the randomized, controlled AgeWell.de study did not lead to higher rates of deprescribing of such drugs by general physicians.

## 1 INTRODUCTION

In older patients with polymedication, the risk of adverse effects is high and age‐ or disease‐related clinical deterioration might be worsened by side effects of drugs.^1^, ^2^ To mitigate risk and avoid potentially harmful treatments, (inter)national lists of potentially inappropriate medications (PIM) for older patients have been developed.^3^ PIMs also include drugs with anticholinergic (side) effects. A study of people aged 75‐90 years found that up to three out of four were taking anticholinergic medication regularly.^4^ In addition, up to 70% of patients with a high anticholinergic burden reported typical side effects such as dry mouth, constipation, near vision problems and urinary problems.^5^, ^6^ Moreover, use of drugs with anticholinergic effects has been associated with long‐term central effects such as cognitive impairment and delirium,^4^ and may be a risk factor for the development of dementia.^5^, ^6^, ^7^, ^8^

It is estimated that in up to 60% of patients taking at least one drug with anticholinergic effects, this medication could generally be discontinued or switched to another drug.^9^ Despite awareness of the risks associated with such medications and the availability of alternative treatments, deprescribing, ie, the planned, gradual withdrawal of drugs, including drugs with anticholinergic effects, remains a challenge due to its time‐consuming and complex nature.^10^, ^11^ Often, the immediate subjective benefit to the patient is small and may be offset by unpleasant withdrawal symptoms.^11^ On the other hand, a drug listed as a PIM could be assessed as actually appropriate in individual cases, considering short‐term effects on a patient's wellbeing, previous experience with other drugs in the patient and the unavailability of more suitable alternatives. A number of studies have shown that an individualized assessment of the benefits and risks for the patient, comprehensive patient information about deprescribing procedures and ongoing motivation during the process facilitate successful and sustained implementation of deprescribing.^11^, ^12^

However, there is limited understanding of the effectiveness of deprescribing drugs with anticholinergic effects and the extent to which deprescribing strategies are prioritized over other interventions to improve care for older patients,^13^, ^14^, ^15^, ^16^ therefore there is a need to identify additional facilitators and barriers impeding deprescribing approaches. This study focuses on the area of deprescribing drugs with anticholinergic effects in older patients in primary care outpatient settings. The aim of the present analysis was to assess the effectiveness of counselling on drug risks as part of a multimodal intervention to prevent cognitive decline, and to examine under which circumstances general practitioners (GPs) have implemented recommendations to reduce prescribing of drugs with anticholinergic effects, as provided in the AgeWell.de study.

## 2 METHODS

The data for the present analysis were collected as part of the AgeWell.de study, a multicentre, cluster‐randomized, controlled, multicomponent intervention study conducted between June 2018 and January 2022 in 123 general practices in Germany.^17^ AgeWell.de aimed to evaluate the effectiveness of a multicomponent intervention programme to prevent or delay cognitive decline in older general practice patients at an increased risk of dementia and was conducted over a 2‐year follow‐up period.^17^, ^18^ The study included participants aged between 60 and 77 years, living at home, with an increased risk of developing dementia (CAIDE Dementia Risk Score ≥9).^17^ The recruitment of the patients took place in the local general practices. GPs were blinded to their group allocation. The main exclusion criteria were pre‐existing conditions that might prevent active participation in the study, pre‐existing or baseline‐diagnosed dementia, as well as severe communication impairments. The multicomponent intervention programme included nutritional counselling, tasks to increase physical and social activity, cognitive training, additional support in case of experiences such as grief, loss and depressive symptoms, management of cardiovascular risk factors, and medication optimization. Participants in the control practices received regular care from their GP as well as general health advice at the baseline interview.

### 2.1 Medication optimization

For the intervention component medication optimization, patient data and medication records were screened for various medication risks. Study nurses from the respective study centres collected patient and medication data in face‐to‐face visits (in both groups) at the beginning of the study (t0) and after two years (t2). The screening on medications risks was performed at the Heidelberg site and included algorithm‐based checking for contraindications due to renal impairment or drug‐drug interactions, potentially missing medications (according to selected START‐STOPP criteria^19^, ^20^), problems with medication therapy management^17^ and drugs with anticholinergic effects. To this end, the screening was performed according to the Heidelberg Anticholinergic Drug List.^21^ In intervention practices, the results of the medication assessment as well as individual recommendations were communicated after the baseline assessment (t0) in a structured letter written in Heidelberg and transferred to the GPs by the study nurses. The letters included an additional form for the GP to complete and return if at least one recommendation was given. This form asked them to indicate whether the recommendations on drug therapy were considered helpful and to explain if and why certain recommendations were or were not implemented by the GP. To maintain the blinding, control practices also received a letter stating that no medication risks were found. For ethical reasons, potential contraindications in the patients' medication were still transmitted. After 24 months (t2), ie, the end of the AgeWell.de study, patient data and medication records were screened again for medication risks using the same methods.

The Heidelberg Anticholinergic Drug List contains 75 active substances, of which 39 are classified as strong (eg, clozapine) and 46 as weak (eg, quetiapine) anticholinergics.^21^ The list has been developed considering drugs on the German pharmaceutical market and includes only drugs with convincing evidence for potentially relevant anticholinergic effects in humans. In brief, the listed drugs had to be systemically active, able to cross the blood‐brain barrier and their anticholinergic activity had to be confirmed by evaluation of the mechanism of action (binding to muscarinic receptors), determination of serum anticholinergic activity [SAA] and/or reported typical anticholinergic effects. In addition to the identification and classification of drugs with anticholinergic effects, an algorithm was developed to provide recommendations (discontinuation, substitution or dose reduction). This list was pilot‐tested in 16 geriatric patients.^21^ Recommendations of specific drugs as possible substitutes for drugs with anticholinergic effects were accompanied by information on drug‐drug interactions (of the potential substitutes with other drugs of a patient) and dosing advice in case of renal impairment.

### 2.2 Data collection

Data were collected in four categories during the 2‐year follow‐up period: specific patient data (eg, age, existing diagnoses, HbA1c levels, serum creatinine, current weight), medication data (eg, drug names, Pharmazentralnummer [PZN], a unique identification code used in Germany for pharmaceutical products), dosage, medication risks identified by the algorithm‐based screening (“reported alerts”) and a survey of the anticholinergic‐related symptoms of constipation, dry mouth and urinary problems in a sense of difficulties in starting urination and/or emptying the bladder. In the intervention group, data were collected during face‐to‐face visits at t0, at 12 months (t1) and at t2. In the control group, data collection took place at t0 and t2.^17^ The present analyses consider the points in time t0 and t2 for better comparability. Based on the specific patient data available at t0 and t2, the total number of patients who completed the intervention component during the 2‐year follow‐up period was determined.

### 2.3 Data analysis

The prevalence of anticholinergic prescriptions overall and per patient at t0 and t2 was first compared between the intervention group and control group (with regard to the use or non‐use of drugs with anticholinergic effects; use was defined as at least one weak or strong anticholinergic). When comparing drugs with anticholinergic effects between t0 and t2, cases were categorized in one of six categories (identical anticholinergics, replaced anticholinergics, decreased number of anticholinergics, increased number of anticholinergics, stopped all anticholinergics and newly started anticholinergics), therefore we first analysed whether patients with an anticholinergic prescription at t0 still received drugs with anticholinergic effects at t2. Thereby, patients receiving drugs with anticholinergic effects at t2 were differentiated between those who were taking drugs with anticholinergic effects at t0 and t2 (“continued exposure”, ie, ongoing anticholinergic exposure, including identical anticholinergics, replaced anticholinergics and lower or higher number of drugs with anticholinergic effects at t0 and t2) and those who had discontinued drugs with anticholinergic effects entirely at t2 (“stopped exposure”). Moreover, we also assessed how many patients received anticholinergic prescriptions only at t2 and not at t0 (“newly started”).

The extent and type of typical anticholinergic symptoms in the two groups at different times were also analysed (categorizing a symptom as not present or present, including mild and severe symptoms). In addition, factors potentially influencing the reduction of drugs with anticholinergic effects over time were examined. Possible influencing factors included the total number of drugs per patient and the number of drugs with anticholinergic effects per patient at t0, the presence of typical anticholinergic symptoms at t0, the patient's group assignment, sex and age, as well as the presence of other identified medication risks.

### 2.4 Statistical analysis

Medication and use of drugs with anticholinergic effects were analysed descriptively (frequencies, means, median, 25th and 75th percentiles). The presence of anticholinergic symptoms was also analysed descriptively (frequencies, means) at the patient level. The chi‐square test for nominally independent samples was used to compare expected and observed frequencies and McNemar’ test was used for nominal dependent samples, ie, McNemar's test was performed to assess whether there was a difference in the occurrence of anticholinergic prescriptions between t0 and t2 within each group (intervention and control). Furthermore, the course of potentially anticholinergic‐related symptoms was analysed using statistical tests.

In addition, it was calculated whether there was a difference between the two groups regarding the presence of symptoms (chi‐square test). Chi‐square tests were also used to assess whether discontinuation behaviour (defined as having an anticholinergic prescription at t0 but not at t2) differed between patients who had at least one anticholinergic‐related symptom at t0. The additional identified medication risks were also evaluated descriptively (frequencies, means) to capture the distribution of these data over a 2‐year period. Subsequently, multivariate logistic regression was used to analyse possible associations and confounders. A significance level of *P* < 0.05 was used for all analyses.

The dependent variable was whether potentially inappropriate anticholinergic prescriptions were discontinued, ie, “stopped exposure”.

Using multivariate logistic regression, we modelled the probability of deprescribing all drugs with anticholinergic effects of a patient depending on the variables patient sex (nominal), patient age (metric), patient group assignment (nominal), number of other drugs (metric), number of other drugs with anticholinergic effects (metric), number of other medication risks (metric) and number of anticholinergic‐related symptoms (metric).

## 3 RESULTS

The AgeWell.de study included 1030 patients, 819 of whom completed the 2‐year follow‐up period.^17^ A total of 808 patients, age 69.2 ± 4.89 years (mean ± standard deviation), 424 (52.5%) females and 384 (47.5%) males (374 in the intervention group, age 69.2 ± 4.86 years, 197 [52.7%] females, 177 [47.3%] males, and 434 in the control group, age 69.2 ± 4.92 years, 227 [52.3%] females, 207 [47.7%] males), were included in the medication optimization analysis. For the remaining 11 patients, required patient data for t0 and t2 were not available and these patients were therefore excluded from the analysis.

### 3.1 General medication data

A total of 368 patients (98.4%) in the intervention group and 420 patients (96.8%) in the control group were taking at least one medication at t0. At t2, 352 patients (94.1%) in the intervention group and 411 patients (94.7%) in the control group were taking at least one medication. The absolute proportion of patients in the intervention group who had discontinued all medications was not significantly different between the intervention and control group (4.3% *vs* 2.1%, *P* = 0.078, chi‐square).

At t0, patients in the intervention group and in the control group had a median of five medications (intervention group: quartil [q]1 = 3, q2 = 5, q3 = 8, minimum = 0, maximum = 21; control group q1 = 3, q2 = 5, q3 = 8, minimum = 0, maximum = 23). At t2, there was no statistically significant change in the median number of medications in either the intervention group (median = 5, q1 = 4, q2 = 5, q3 = 8, *P* = 0.979 [McNemar's test]) or the control group (median = 5, q1 = 3, q2 = 5, q3 = 8, *P* = 0.862 [McNemar's test]).

### 3.2 Medication risks

Overall, 140 patients (37.4%) in the intervention group and 139 patients (32%) in the control group had at least one identified potential medication risk (reported alert) at t0 (*P* = 0.107, chi‐square test) (ie, any medication risk analysed in the AgeWell.de study, including drugs with anticholinergic effects; Table 1). After 2 years, at least one medication risk was identified in 125 patients (35.3%) in the intervention group and in 131 patients (31.6%) in the control group (*P* = 0.251, chi‐square test; Table 2). No significant differences in the occurrence of medication risks between t0 and t2 were found within each group (intervention group *P* = 0.213, control group *P* = 0.488, McNemar's test).

**TABLE 1:** Number of identified potential medication risks at baseline (t0).

Subgroup	Medication risks					
	Patients with at least one (additional) medication risk	Prescription of drugs with anticholinergic effects	Contraindication due to drug‐drug interaction	Contraindication due to renal impairment	START criteria	Patient reported medication problems
IG	140 (37.4%)	71 (19.3%)	1 (0.3%)	2 (0.5%)	77 (20.9%)	9 (2.4%)
CG	139 (32%)	61 (14.5%)	0	0	82 (19.5%)	19 (4.5%)
IG with drugs with anticholinergic effects	17 (23.9%)	71 (100%)	1 (1.4%)	0 (0%)	12 (16.9%)	5 (7.0%)
CG with drugs with anticholinergic effects	18 (29.5%)	61 (100%)	0 (0%)	0 (0%)	15 (24.6%)	3 (4.9%)

Abbreviations: CG, control group, N = 434; IG, intervention group, N = 374.

**TABLE 2:** Number of identified potential medication risks at follow‐up (t2). IG = intervention group, CG = control group.

Subgroup	Medication risks					
	Patients with at least one (additional) medication risk	Prescription of drugs with anticholinergic effects	Contraindication due to drug‐drug interaction	Contraindication due to renal impairment	START criteria	Patient reported medication problems
IG	125 (35.3%)	63 (17.9%)	3 (0.9%)	3 (0.9%)	69 (19.5%)	3 (0.9%)
CG	131 (31.6%)	59 (14.4%)	1 (0.2%)	3 (0.7%)	88 (21.3%)	4 (1%)
IG with drugs with anticholinergic effects (overall)	14 (22.2%)	63 (100%)	2 (3.2%)	0 (0%)	11 (17.5%)	1 (1.6%)
CG with drugs with anticholinergic effects (overall)	22 (37.3%)	59 (100%)	1 (1.7%)	0 (0%)	21 (35.6%)	0 (0%)
Patients with drugs with anticholinergic effects at t0 (continued exposure)	25 (27.2%)	92 (100%)	3 (3.3%)	0 (0%)	21 (22.8%)	1 (1.1%)
IG (continued exposure)	11 (22.9%)	48 (100%)	2 (4.2%)	0 (0%)	8 (16. 7%)	1 (2.1%)
CG (continued exposure)	14 (31.8%)	44 (100%)	1 (2.3%)	0 (0%)	13 (29.5%)	0 (0%)
Patients with drugs with anticholinergic effects at t0 (stopped exposure)	6 (15%)	‐	0 (0%)	0 (0%)	6 (15%)	0 (0%)
IG (stopped exposure)	1 (4.3%)	‐	0 (0%)	0 (0%)	1 (4.3%)	0 (0%)
CG (stopped exposure)	5 (29.4%)	‐	0 (0%)	0 (0%)	5 (29.4%)	0 (0%)
Patients without drugs with anticholinergic effects at t0 (new exposure)	11 (7.7%)	30 (100%)	0 (0%)	0 (0%)	11 (8.5%)	0 (0%)
IG (new exposure)	3 (20.0%)	15 (100%)	0 (0%)	0 (0%)	3 (5.0%)	0 (0%)
CG (new exposure)	8 (53.3%)	15 (100%)	0 (0%)	0 (0%)	8 (11.4%)	0 (0%)

Abbreviations: CG, control group; IG, intervention group.

At t0, 132 patients received at least one anticholinergic prescription (intervention group 71 patients (19.3%); control group 61 patients (14.5%); *P* = 0.074; chi‐square test; Table 1). Table 3 provides an overview of the prescribed drugs with anticholinergic effects and their frequency. When looking at the overall anticholinergic exposure at t2 (ie, continued and newly‐started), 63 patients in the intervention group (17.9%) and 59 patients (14.4%) in the control group (*P* = 0.183, chi‐square test) received at least one drug with anticholinergic effects (Table 2). There was no significant difference between the two groups in the presence of drugs with anticholinergic effects at t0 and t2.

**TABLE 3:** Drugs with anticholinergic effects at baseline (t0) in the intervention group, classified as strong or weak, with feedback(s) from the GP.

ATC classification	Drug	Strong	Weak	Feedback from the GP
Alimentary tract and metabolism	Butylscopolamine (n = 1)		x	‐
	Butylscopolamine and paracetamol (acetaminophen) (n = 1)		x	“The medication was discontinued.”
	Loperamide (n = 2)		x	“The medication was discontinued. It was not prescribed by us; it may have been obtained by the patient independently.” “The patient refuses deprescribing.”
Genito‐urinary system and sex hormones	Oxybutynin (n = 1)	x		“Any adjustment and monitoring must be carried out by a specialist. Following urological assessment, a change of medication was not recommended.”
	Propiverine (n = 3)		x	“Any adjustment and monitoring must be carried out by a specialist.” (n = 3)
	Solifenacin (n = 6)	x		“The medication was changed to trospium.” “Any adjustment and monitoring must be carried out by a specialist.” “The change in medication is being carried out by the urologist.” “The patient refuses deprescribing.” (n = 2)
	Tolterodine (n = 1)	x		“Any adjustment and monitoring must be carried out by a specialist.”
	Trospium (n = 5)		x	“The medication was changed to finasteride.” “Any adjustment and monitoring must be carried out by a specialist.” (n = 2) “The medication was discontinued.” “The medication was prescribed by the gynaecologist.”
Nervous system	Amitriptyline (n = 5)	x		“No changes were made (no reasons provided).” “The medication is used as migraine prophylaxis and was prescribed by the pain specialist. The medication is very effective.” “The medication was prescribed only for short‐term use as needed.” “The patient stopped taking the medication on his own.” “The medication was prescribed by a specialist and is being monitored by them. Discontinuation is tentatively planned.”
	Biperiden (n = 1)	x		“This is a prescription provided by a specialist.”
	Buprenorphine (n = 1)		x	“The patient had already taken the recommended alternatives, which were either ineffective or not well tolerated. These included ibuprofen and metamizole.”
	Cinnarizine and dimenhydrinate (n = 3)	x		“The patient takes the medication only as needed for morning dizziness.” “The patient refuses deprescribing.” “No changes were made (no reasons provided).”
	Codeine and paracetamol (acetaminophen) (n = 1)		x	“The patient occasionally takes a tablet for certain headaches and does not wish to switch medications.”
	Diazepam (n = 4)		x	“The patient refuses deprescribing.” (n = 2) “The patient is fixated on the medication; dependence is likely.”
	Doxepin (n = 3)	x		“The medication was changed to sertraline.” “The medication was discontinued.” “The patient refuses deprescribing.”
	Levomepromazine (n = 1)	x		“The patient refuses deprescribing.”
	Opipramol (n = 1)		x	“The medication was changed to sertraline.”
	Oxycodone (n = 3)		x	“The dosage was decreased.” “The patient refuses deprescribing.” (n = 2)
	Oxycodone and naloxone (n = 2)		x	“No clinically appropriate alternative is available.” “The neurologist prescribed the medication and is monitoring the therapy.”
	Paracetamol (acetaminophen) and chlorphenamine (n = 1)	x		“The therapy was administered for 4 days, after which the medication was discontinued.”
	Paroxetine (n = 2)		x	‐
	Perazine (n = 1)		x	“The medication was prescribed by a specialist and is being monitored by him. Discontinuation is tentatively planned.”
	Promethazine (n = 2)		x	“The patient refuses deprescribing.” “No changes were made (no reasons provided).”
	Quetiapine (n = 2)	x		“This is a prescription provided by a specialist.” “The patient refuses deprescribing.”
	Sulpiride (n = 2)		x	“The medication was discontinued.” “No changes were made (no reasons provided).”
	Tapentadol (n = 5)		x	“The patient had already taken the recommended alternatives, which were either ineffective or not well tolerated. These included ibuprofen, diclofenac, and tilidine+naloxone.” “The patient had already taken the recommended alternatives, which were either ineffective or not well tolerated. These included tilidine.” “The medication is well tolerated by the patient.” “No changes were made (no reasons provided).”
	Tramadol (n = 3)		x	“The medication was discontinued.” “No changes were made (no reasons provided).” (n = 2)
	Tramadol and paracetamol (acetaminophen) (n = 1)		x	…
	Trimipramine (n = 4)	x		“There is no somnolent alternative.” (n = 2) “The medication was discontinued.” “The patient refuses deprescribing.”
Respiratory system	Aclidinium bromide (n = 1)		x	“Any adjustment and monitoring must be carried out by a specialist.”
	Fenoterol and ipratropium bromide (n = 3)		x	“The medication was discontinued.” “Any adjustment and monitoring must be carried out by a specialist.” (n = 2)
	Formoterol and aclidinium bromide (n = 1)		x	“The dosage of the alternative medication is not sufficiently effective.”
	Glycopyrronium bromide (n = 1)		x	“No deprescribing was performed. The patient has a chronic lung disease. In this indication, no standardized recommendation is possible.”
	Indacaterol and glycopyrronium bromide (n = 2)		x	…
	Olodaterol and tiotropium bromide (n = 1)		x	“The patient refuses deprescribing. He reports that the medication effectively alleviates his dyspnoea and that he has had positive experience with its use.”
	Tiotropium bromide (n = 4)		x	“The patient has significant pulmonary obstruction; the proposed alternatives are not appropriate.” “Any adjustment and monitoring must be carried out by a specialist.” (n = 2) “The medication was discontinued.”
	Vilanterol and umeclidinium bromide (n = 4)		x	“Any adjustment and monitoring must be carried out by a specialist.” (n = 2) “Guideline‐based therapy for COPD.” “The therapy adheres to established clinical guidelines and involves anticholinergic effects for which no alternative treatment options are available.”

Abbreviations: GP, general practitioner.

Hence, the overall proportion of patients with at least one anticholinergic alert in the intervention group did not change significantly over the 2‐year period (*P* = 0.324, McNemar's test). Similarly, the proportion of patients with anticholinergic alerts in the control group did not change over 2 years (*P* = 1.000, McNemar's test).

At t2, in 28 patients with previous anticholinergic alert (39.4%) in the intervention group the originally prescribed drug(s) with anticholinergic effects were discontinued, reduced or replaced compared to 30 patients in the control group (49.2%, *P* = 0.089) (Table 4). There were only seven cases in the “replaced” category, which were not further analysed.

**TABLE 4:** Prescriptions of drugs with anticholinergic effects at follow‐up (t2) in patients with drugs with anticholinergic effects at baseline (t0).

	Prescriptions of drugs with anticholinergic effects at t2				
Subgroup	Discontinued	Reduced	Replaced with a more appropriate drug	Identical drugs with anticholinergic effects	Higher number of drugs with anticholinergic effects
IG (n = 71)	23 (32.3%)	3 (4.2%)	2 (2.8%)	41 (57.7%)	2 (2.8%)
CG (n = 61)	17 (27.9%)	8 (13.1%)	5 (8.2%)	29 (47.5%)	2 (3.3%)
All patients (n = 132)	40 (30.3%)	11 (8.3%)	7 (5.3%)	70 (53.0%)	4 (3.0%)

Abbreviations: CG, control group; IG, intervention group.

### 3.3 Prevalence of patient‐reported anticholinergic symptoms

Of the 132 patients taking drugs with anticholinergic effects at t0, 58 (43.9%) had discontinued, reduced or replaced their drugs with anticholinergic effects at t2. In the “replaced” category, no distinction was made between substituted anticholinergics with weaker and stronger anticholinergic activity. In 74 (50.3%) patients, there was no change in the anticholinergic medication or the number of drugs with anticholinergic effects was increased.

Overall, in patients taking drugs with anticholinergic effects, 74 out of 132 patients reported at least one anticholinergic symptom (56.1%) (Table 5). Of the patients who discontinued, reduced or replaced their anticholinergic medication, 34 (58.6%) had reported at least one anticholinergic symptom at t0, while 24 (41.4%) % had not reported such symptoms (*P* = 0.237). Of the patients who continued or increased their anticholinergic medication, 40 (54.1%) had reported at least one symptom at t0, while 33 (44.6%) had not reported such symptoms (*P* = 0.483). One patient did not provide any information about his symptoms. Thus, patients who discontinued or reduced their anticholinergic medication tended to have reported symptoms more often at t0 than those who continued their anticholinergic medication (58.6 *vs* 54.1%). However, this difference was not statistically significant, *P* = 0.561.

**TABLE 5:** Suspected anticholinergic symptoms at baseline (t0) and follow‐up (t2) (N = t0/t2).

Subgroup	Symptoms											
	At least one anticholinergic symptom			Constipation			Dry mouth			Urinary problems		
	t0	t2	*P* (McNemar)	t0	t2	*P* (McNemar)	t0	t2	*P* (McNemar)	t0	t2	*P* (McNemar)
IG with drugs with anticholinergic effects (n = 71/63)	39 (54.9%)	30 (47.6%)	0.263	14 (19.7%)	7 (11.1%)	0.267	32 (45.1%)	24 (38.1%)	0.302	10 (14.1%)	6 (9.5%)	1.000
CG with drugs with anticholinergic effects (n = 61/59)	35 (57.4%)	28 (47.5%)	0.815	8 (13.1%)	7 (11.9%)	0.1000	28 (45.9%)	25 (42.4%)	1.000	8 (13.1%)	6 (10.2%)	0.754
All patients with drugs with anticholinergic effects (n = 132/122)	74 (56.1%)	58 (47.5%)	0.256	22 (16.7%)	14 (11.5%)	0.454	60 (45.5%)	49 (40.2%)	0.442	18 (13.6%)	12 (9.8%)	0.629
*P* (chi‐square)	0.778	0.986		0.310	0.896		0.924	0.630		0.871	0.905	
IG with medications (Ach excluded) (n = 297/289)	96 (32.3%)	88 (30.6%)	0.268	27 (9.1%)	28 (9.7%)	1.000	70 (23.6%)	59 (20.5%)	0.169	21 (7.1%)	20 (6.9%)	0.839
CG with medications (Ach excluded) (n = 359/352)	126 (35.1%)	120 (34.2%)	0.617	46 (12.8%)	34 (9.7%)	0.072	88 (24.5%)	81 (23.1%)	0.738	25 (7.0%)	29 (8.3%)	1.000
All patients with medications (Ach excluded) (n = 656/641)	222 (33.8%)	208 (32.6%)	0.244	73 (11.1%)	62 (9.7%)	0.165	158 (24.1%)	140 (21.9%)	0.225	46 (7.0%)	49 (7.7%)	1.000
*P* (chi‐square)	0.564	0.295		0.154	0.905		0.881	0.397		0.907	0.511	
IG with medications (n = 368/352)	135 (36.7%)	118 (33.6%)	0.353	41 (11.1%)	35 (10%)	0.551	102 (27.7%)	83 (23.6%)	0.141	31 (8.4%)	26 (7.4%)	0.456
CG with medications (n = 420/=411)	161 (38.3%)	148 (36.1%)	0.430	54 (12.9%)	41 (10%)	0.093	116 (27.6%)	106 (25.9%)	0.282	33 (7.9%)	35 (8.5%)	0.241
All patients with medications (n = 788/763)	296 (37.6%)	264 (34.6%)	0.204	95 (12.5%)	76 (10.0%)	0.098	218 (27.7%)	189 (24.8%)	0.100	64 (8.1%)	61 (8.0%)	0.912
*P* (chi‐square)	0.634	0.475		0.487	0.116		0.645	0.132		0.620	0.195	
IG without medications (n = 622)	0 (0%)	3 (13.6%)	**…**	0 (0%)	0 (0%)	**…**	0 (0%)	0 (0%)	…	0 (0%)	3 (13.6%)	…
CG without medications (n = 1423)	1 (7.1%)	3 (13.0%)	**…**	0 (0%)	1 (4.3%)	**…**	0 (0%)	0 (0%)	…	1 (7.1%)	2 (8.7%)	…
All patients without medications (n = 20/45)	1 (5.0%)	6 (13.3%)	**…**	0 (0%)	1 (2.2%)	**…**	0 (0%)	0 (0%)	…	1 (5.0%)	5 (11.1%)	…
*P* (chi‐square)	…	…		…	…		…	…		…	…	

Abbreviations: Ach = drugs with anticholinergic effects; CG, control group; IG, intervention group.

Overall, when combining control and intervention groups, the incidence of patient‐reported anticholinergic symptoms was higher with medication use. At t0, 1 of 20 patients (5.0%) reported at least one symptom when taking no medication. When taking medication but without drugs with anticholinergic effects, 222 out of 656 patients (33.8%) reported at least one anticholinergic symptom. (Table 5).

### 3.4 Analysis of factors influencing the acceptance of anticholinergic alerts

In the logistic regression analysis none of the investigated predictors had a significant influence on the presence or absence of drugs with anticholinergic effects at t2 in patients who had drugs with anticholinergic effects at t0 (Table 6).

**TABLE 6:** Results of multivariable logistic regression analysis.

Predictor	Coefficient (β)	Standard error (SE)	Wald‐statistic	*P*	Odds ratio (OR)	Confidence interval (95% CI)
Age	0.038	0.043	0.794	0.373	1.039	[0.955‐1.130]
Sex	0.510	0.403	1.596	0.206	1.665	[0.755‐3.670]
Group	−0.351	0.416	0.715	0.398	0.704	[0.312‐1.589]
Number of other medications (t0)	0.059	0.052	1.306	0.253	1.061	[0.958‐1.175]
Number of other drugs with anticholinergic effects (t0)	−0.639	0.463	1.906	0.167	0.528	[0.213‐1.308]
Number of other medication risks (t0)	0.283	0.284	0.993	0.319	1.327	[0.761‐2.313]
Number of anticholinergic symptoms (t0)	−0.039	0.260	0.022	0.881	0.962	[0.578‐1.602]

### 3.5 Feedback from general practitioners

A total of 161 responses regarding the identified medication risks and provided recommendations were send back from the GPs, of which 74 (46.0%) concerned the risk of a potentially inappropriate anticholinergic prescription. In 48 of these cases (64.9%), no changes were made, mostly due to so‐called “other”, not prespecified, reasons (38.4%) such as acute pain situations, known intolerances to alternative drugs or apparently good tolerance of the current medication. Additional reasons for not changing drugs with anticholinergic effects included the need for another specialist (eg, psychiatrist) to make the recommended change (34.6%), for example in case of antidepressants and antipsychotics, patients refusing the recommended change (11.6%) or suggested alternatives being deemed ineffective or inappropriate for other reasons (7.7%) (Table 3).

## 4 DISCUSSION

In the present study we analysed drugs with anticholinergic effects in an outpatient group of patients at increased risk of dementia participating in the AgeWell.de study. In the intervention group, the attending GPs were informed about the prescription of drugs with anticholinergic effects at the beginning of the study and received written recommendations to reduce such drugs in individual patients.

The number of patients taking at least one drug with anticholinergic effects decreased in both groups over the 2 years, yet this decrease was not statistically significant nor was there a difference between the intervention and the control group. Similarly, the number of patients on drug with anticholinergic effects remained comparable over time because of newly prescribed drug with anticholinergic effects.

Anticholinergic symptoms were reported more often if patients were taking medication, particularly drugs with anticholinergic effects. In patients who were taking drugs with anticholinergic effects and reported symptoms, these drugs were discontinued numerically more often, but the difference was not statistically significant compared to patients without symptoms. Nevertheless, these data suggest that the use of drugs with anticholinergic effects may be associated with a higher incidence of symptoms and that measures to deprescribe might be implemented more often when such symptoms are present. No significant differences were found between the intervention and control groups. This suggests that either the intervention in the AgeWell.de study was not very effective in achieving the goal of deprescribing drugs with anticholinergic effects and/or it was attenuated by a general trend to reduce drugs with anticholinergic effects in the control group, possibly because of an increased awareness of the GPs generally or triggered by participation in the AgeWell.de study and by receiving the structured letter, even if it did not contain any recommendations.

These results suggest that the recommendations given to optimize medication—particularly with regard to the deprescribing of drugs with anticholinergic effects—did not trigger a widespread reduction of anticholinergic prescribing. This confirms the findings of previous studies that reducing polypharmacy, especially with medications such as drugs with anticholinergic effects, is a complex task.^22^

When examining the reasons why recommended deprescribing of drugs with anticholinergic effects was not carried out despite evidence for existing risks, it becomes clear that these reasons often relate to the individual clinical situation of the patient. On the other hand, it was frequently noted that, in the GPs view, the responsibility for deprescribing a specific drug would lie with the prescribing specialists, a finding consistent with previous studies.^23^ Thus, the GPs might be reluctant to make changes because they fear potentially negative consequences for their patients, which they cannot adequately assess themselves,^24^ and because they lack the time to obtain advice from the specialist. Therefore, it could have been beneficial to inform the patient's specialists about the medication risks, too. At the same time, the response letters from the GPs also suggest that an acute need for the medication, apparently good tolerability and therefore presumed absence of risks may be reasons for not deprescribing a drug with anticholinergic effects. However, it is questionable whether a good tolerance is a predictor of dementia risks. Previous studies have shown that low motivation due to a lack of empowerment,^25^ insufficient system‐related resources and the complex organization of deprescribing processes are limiting factors for switching or discontinuing anticholinergics.^26^, ^27^ At the same time, it is known that a trusting relationship with patients and good communication,^28^ as well as the involvement of other healthcare professionals and repeated intervention, are important facilitators in the deprescribing processes.^26^ Moreover, the success of deprescribing efforts also depends on the targeted patient population and the availability of appropriate therapeutic alternatives.

The medication optimisation intervention, including the deprescribing of drugs with anticholinergic effects, was one of several complex intervention components in the AgeWell.de study. While the feedback received from physicians suggested that general risks with drugs with anticholinergic effects were understood, the low numbers of actual deprescribing attempts indicate that key factors were lacking to enable sustainable and successful deprescribing in individual cases. This might be explained by the fact that success factors known from previous studies, such as the involvement of different professional groups, the education and motivation of patients about the benefits of deprescribing or repetitive follow‐ups on deprescribing suggestions, were not addressed intensively enough in the intervention design, likely because the medication optimization intervention was just one amongst many other resource‐intensive interventions. As a measure to increase awareness, the GPs in the intervention practices received written information on the risks of drugs with anticholinergic effects with regard to dementia at the beginning of the AgeWell.de study. Unfortunately, more intensive education of the GPs was not within the scope of the project, which aimed to develop and study a multicomponent intervention that should be conveyed largely by study nurses. Repeated screening and provision of recommendations on medication risks to the GPs during the course of the study was not possible because of financial reasons. Direct communication of staff members from Heidelberg, the responsible site for the medication optimization intervention, with patients or their specialists was not possible because of privacy reasons and the fear that independent recommendations on drug therapy given directly to the patients could be detrimental to the physician‐patient relationship. This suggests, however, that the complexity of deprescribing drugs with anticholinergic effects requires a comprehensive and multimodal intervention itself.

The study has some limitations, both in terms of the design as well as the area of data collection and management. First and foremost, the design of the study was not intended to specifically promote deprescribing of drugs with anticholinergic effects, but rather to address a total of five distinct medication risks and provide one‐time recommendations after initial assessment (otherwise GPs were not specifically informed to avoid drugs with anticholinergic effects during the trial). Yet, this offers the opportunity to assess how general recommendations on deprescribing are dealt with in the context of many other recommendations to improve care. Second, the study participants were selected from older adults with an increased risk of dementia. As there was no specific indication for drugs with anticholinergic effects as an inclusion criterion, only about one out of six participants were taking at least one drug with anticholinergic effects at the start of the study. This small number may have obscured effects of the intervention. Additionally, no clinically validated anticholinergic scale was used to formally assess the anticholinergic burden by calculating a summary score. This was primarily because the focus was on the multimodal intervention design and a score would not necessarily have meaningfully complemented the clinical evaluation. Furthermore, the study involved a specific patient group (with an increased risk of dementia and on average experiencing polypharmacy). Many of the existing scales are not validated for this patient population, which limits their interpretive value in this context. Similarly, the Heidelberg List is not validated for calculating a score of anticholinergic burden.

In summary, the medication optimisation intervention in the AgeWell.de study did not lead to a significant reduction of drugs with anticholinergic effects compared to the control group. This confirms findings from previous studies and suggests that patients may have needed closer and more sustained attendance to deprescribe potentially inappropriate anticholinergic medication. Further studies are needed to determine how to achieve sustained effectiveness in reducing the prescription of these drugs. The analysis of various predictors of deprescribing behaviour showed no significance. Therefore, this study suggests that the decision to discontinue a drug with anticholinergic effects of concern did not depend on study‐related factors, although the sample size did not allow for definitive conclusions.

## AUTHOR CONTRIBUTIONS

SRH and DC: Conceptualization, supervision. AP, ML, JD, MW, TF, JG, WH, HK, HHK, JRT and BW: Support in conceptualization and study design. LKL, HMS, SRH and DC: Methodology. LKL and BW: Data curation. LKL and HMS: Formal analysis, writing—original draft. HMS and DC: Project administration. All authors: Writing—review and editing. All authors have read and approved the final version of the manuscript.

## CONFLICT OF INTEREST STATEMENT

The authors declare that they have no conflict of interest.

## CLINICAL TRIAL REGISTRATION

DRKS00013555.

## Data Availability

The data underlying the current study are not publicly available due to privacy restrictions. Data are available after de‐identification to researchers who submit a sound proposal to the AgeWell.de steering committee. Respective requests should be submitted to steffi.riedel-heller@medizin.uni-leipzig.de.
